# CP204L Is a Multifunctional Protein of African Swine Fever Virus That Interacts with the VPS39 Subunit of the Homotypic Fusion and Vacuole Protein Sorting Complex and Promotes Lysosome Clustering

**DOI:** 10.1128/jvi.01943-22

**Published:** 2023-02-01

**Authors:** Katarzyna Magdalena Dolata, Walter Fuchs, Grégory Caignard, Juliette Dupré, Katrin Pannhorst, Sandra Blome, Thomas C. Mettenleiter, Axel Karger

**Affiliations:** a Institute of Molecular Virology and Cell Biology, Friedrich-Loeffler-Institut, Greifswald-Insel Riems, Germany; b UMR Virologie, INRAE, Ecole Nationale Vétérinaire d’Alfort, Laboratoire de Santé Animale d’Alfort, Anses, Université Paris-Est, Maisons-Alfort, France; c Institute of Diagnostic Virology, Friedrich-Loeffler-Institut, Greifswald-Insel Riems, Germany; Northwestern University Feinberg School of Medicine

**Keywords:** African swine fever virus, ASFV, CP204L, VPS39, HOPS complex, virus-host interaction, lysosomes

## Abstract

Virus replication depends on a complex interplay between viral and host proteins. In the case of African swine fever virus (ASFV), a large DNA virus, only a few virus-host protein-protein interactions have been identified to date. In this study, we demonstrate that the ASFV protein CP204L interacts with the cellular homotypic fusion and protein sorting (HOPS) protein VPS39, blocking its association with the lysosomal HOPS complex, which modulates endolysosomal trafficking and promotes lysosome clustering. Instead, CP204L and VPS39 are targeted to virus factories and localized at the periphery of the virus DNA replication sites. Furthermore, we show that loss of VPS39 reduces the levels of virus proteins synthesized in the early phase of infection and delays ASFV replication but does not completely inhibit it. Collectively, these results identify a novel virus-host protein interaction that modulates host membrane rearrangement during infection and provide evidence that CP204L is a multifunctional protein engaged in distinct steps of the ASFV life cycle.

**IMPORTANCE** African swine fever virus (ASFV) was first identified over a hundred years ago. Since then, much effort has been made to understand the pathogenesis of ASFV. However, the specific roles of many individual ASFV proteins during the infection remain enigmatic. This study provides evidence that CP204L, one of the most abundant ASFV proteins, modulates endosomal trafficking during virus infection. Through protein-protein interaction, CP204L prevents the recruitment of VPS39 to the endosomal and lysosomal membranes, resulting in their accumulation. Consequently, CP204L and VPS39 become sequestered in the ASFV replication and assembly site, known as the virus factory. These results uncover a novel function of viral protein CP204L and extend our understanding of complex interaction between virus and host.

## INTRODUCTION

African swine fever virus (ASFV) causes a contagious and often lethal disease of domestic pigs and wild boars. The disease was first reported in Kenya in 1921 (1) and has remained endemic in Africa. Over the years, sporadic outbreaks were registered outside Africa, but only in 2019 did African swine fever (ASF) reach a pandemic level. There has been no evidence so far of cross-species transmission of ASFV to humans or mammals other than members of the family *Suidae*. Nevertheless, due to high case fatality rates approaching 100% in Eurasian suids and the lack of vaccines, the economic consequences of ASF are very high (2–4).

ASFV is a large double-stranded DNA virus of the family *Asfarviridae* that induces the synthesis of over 100 virus proteins in infected cells (5, 6). About 68 proteins are incorporated into virions (7). The virus replicates mainly in porcine monocytes and macrophages, although other cell types can be infected, especially in the later stages of the disease (8, 9).

ASFV utilizes multiple strategies to enter the host cell, including (i) binding to a hitherto-unknown receptor followed by clathrin-mediated endocytosis (10), (ii) macropinocytosis (11), and (iii) phagocytosis (12). Once internalized, viral particles are trafficked along the endocytic pathway from peripheral early endosomes to late perinuclear endosomes (13). The acidic environment in late endosomes destabilizes the outer viral capsid and exposes its inner membrane, allowing the virus to fuse with the endosomal membrane and release the viral core with the genomic DNA into the cytoplasm (14, 15).

Like several other large DNA viruses, such as poxviruses and iridoviruses, the replication of ASFV is associated with cytoplasmic foci, referred to as virus factories (VFs). ASFV-induced VFs appear as complex and dynamic perinuclear structures close to the microtubule organizing center, surrounded by mitochondria (16) and a vimentin cage (17). ASFV factories are highly compartmentalized to coordinate different steps of the viral life cycle, such as virus DNA replication, transcription and translation, and virion assembly (16, 18). Importantly, VF compartmentalization may protect the virus from degradation by antiviral defense mechanisms of the host cell. Despite their importance, the morphology of VFs and the mechanisms that lead to changes in the cellular organization required to produce complex replication sites are not yet understood.

It is known that ASFV reorganizes endosomal trafficking for its journey toward the perinuclear site, but endosomal membranes are also recruited to early VFs (19). The exact role of endosomal components in ASFV assembly is unknown. It has been suggested that endosomal compartments could be required for virus replication by providing a scaffold and confining the replication process to a specific cytoplasmic location. On the other hand, endosomal membranes may serve as intermediates for virus assembly. The virus-host interaction that leads to endosome accumulation in the early VF must occur in the initial phase of infection (13). Thus, characterizing interactions between host proteins and ASFV early proteins, synthesized before viral DNA replication, could shed light on the mechanism of VF assembly and transport of endosomal membranes into the factory.

The *CP204L* gene is conserved in all ASFV isolates and encodes a highly antigenic viral protein (20) essential for viral replication (21). The CP204L protein (referred to here as CP204L), also known as P30 or P32, is one of the most abundant viral proteins synthesized early during infection (22–24). In infected cells, CP204L is mainly localized in the cytoplasm, but small amounts have also been detected in the nucleus and at the plasma membrane (25). The nuclear CP204L has been reported to interact with the heterogeneous nuclear ribonucleoprotein K (hnRNP-K) (26). A recent study by Chen et al. (27) identified seven cellular proteins interacting with CP204L, which are involved in endocytosis and innate immune response. However, the molecular mechanisms by which CP204L interacts with the host and influences virus replication remain unexplored.

This study identified a set of novel cellular and viral protein interactors of ASFV CP204L. In particular, we focused on host vesicular trafficking proteins, which are the key factors mediating ASFV infection progression. We demonstrate that CP204L interacts with VPS39, a component of the homotypic fusion and vacuole protein sorting (HOPS) complex, blocking VPS39 attachment to the HOPS complex and causing lysosome clustering. We discovered that CP204L is recruited to VFs at early times during infection. Moreover, our observations suggest that VPS39 is an important host factor that regulates the early steps of infection but is not essential for ASFV replication.

## RESULTS

### Identification of the ASFV CP204L interactome.

**FIG 1:**
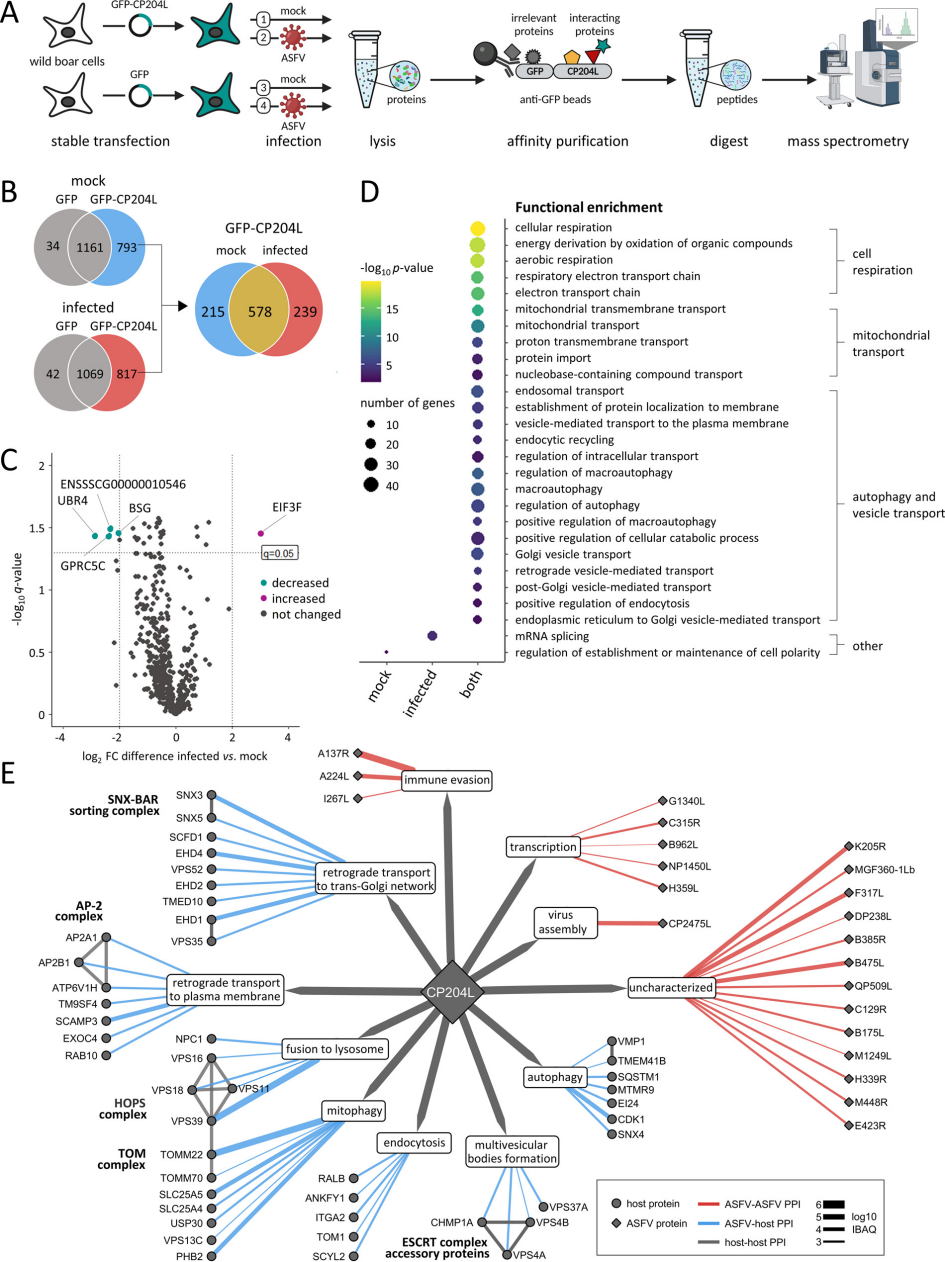
Identification of the ASFV CP204L interactome. (A) AP-MS experimental workflow for identifying interactions between CP204L and host proteins. (B) Venn diagrams demonstrating the overlap between protein interaction partners identified under different conditions. Venn diagrams on the left show GFP background proteins (false-positive hits) (gray) and CP204L-specific proteins identified under two conditions: mock infection (blue) and ASFV infection (red). GFP background proteins were excluded from the list of interacting proteins. The interactors of CP204L identified in mock-infected and virus-infected WSL cells overlap in the Venn diagram (yellow) on the right. (C) Volcano plot showing differences in the abundance of CP204L host protein interactors identified in mock-infected and ASFV-infected samples. The *x* axis represents fold change (FC; infected versus mock infected) on a log_2_ scale. Dotted vertical and horizontal lines indicate the chosen cutoffs for fold changes [|log_2_(FC)| > 2] and *q* values (*q *< 0.05), respectively. Proteins showing significant differences in abundance between mock-infected and virus-infected cells are marked by their gene names. The terms “increased” and “decreased” refer to the indicated proteins with increased or decreased abundances in the mock-infected sample. (D) Selected functional GO terms from overrepresentation analysis are shown for each data set of CP204L interactors: 215 proteins from noninfected (“mock”) samples, 239 from ASFV-infected cells (“infected”), and 578 common proteins from noninfected and ASFV-infected cells (“both”). The most enriched terms are related to cell respiration, mitochondrial transport, autophagy, vesicle transport, and others. The color scale indicates significance, expressed as −log_10_ *P* value, and the size of the dots reflects the number of input genes associated with the respective GO term. (E) Network illustrating the interactions of CP204L with other ASFV proteins and host proteins involved in vesicle transport in the cell. Each node represents a protein (circles, host proteins; diamonds, ASFV proteins). Each edge is colored according to the type of interaction (blue, ASFV-host PPIs; red, ASFV-ASFV PPIs). Edge thickness is proportional to the log_10_ iBAQ value. Physical interactions among host proteins and their specific cellular functions were curated from the literature (Data Set S1C). All CP204L-host interactions shown were identified in mock-infected and virus-infected cells. CP204L-virus interactions were identified only in infected cells.

To gain insight into the host protein interactome of CP204L in ASFV-infected and noninfected cells, we employed an affinity tag purification-mass spectrometry (AP-MS) approach (Fig. 1A). We included AP-MS experiments with infected cells to identify interactions of CP204L with other viral proteins and interactions with host proteins that depend on the presence of other viral proteins or on other conditions found exclusively in the infected cell. The CP204L of the highly virulent ASFV Georgia (28) with a C-terminal green fluorescent protein (GFP) tag was used as bait. The protein was stably expressed in wild boar lung (WSL) cells. GFP-expressing WSL cells were used as a negative control. Proteins were expressed under the following conditions: (i) CP204L-GFP without infection (mock), (ii) CP204L-GFP with ASFV infection, (iii) GFP without infection, and (iv) GFP with ASFV infection. Proteins were affinity purified in biological quadruplicates except for GFP without infection proteins, which was prepared in triplicate. Next, samples were subjected to analysis by mass spectrometry to identify copurifying partners. To minimize the false-positive identifications, host proteins bound to GFP in the absence of bait protein CP204L were excluded from further analysis (Fig. 1B).

Comparing the identified cellular interactors of the CP204L in infected and mock-infected cells revealed an overlap of 578 interactions. Additionally, 239 copurified proteins were identified exclusively in virus-infected cells and 215 in cells without virus infection (see all identified proteins in Data Set S1A in the supplemental material). Among the 578 overlapping proteins, only five showed significant changes in protein levels after infection (Fig. 1C).

To further functionally characterize the CP204L copurifying proteins, we performed a Gene Ontology (GO) enrichment analysis via the traditional overrepresentation statistical method (Fig. 1D). The enrichment analysis of proteins identified exclusively in mock-infected and infected cells revealed their association with mRNA splicing and regulation of cell polarity, respectively. Nevertheless, most enriched proteins were found in the overlapping group, and those robust interactions were analyzed further, as they are more likely to be functionally important. Proteins interacting with the CP204L in mock-infected and infected cells were enriched for several broad terms, such as cellular respiration, mitochondrial transport, and vesicle-mediated transport (Data Set S1B). The latter category included a set of proteins involved in cellular processes critical for virus entry, immune evasion, and cell-to-cell spread, like endocytosis, autophagy, or retrograde trafficking. We therefore constructed a protein interaction subnetwork and looked specifically into interactions between CP204L and host proteins involved in vesicle transport, as well as interactions between CP204L and other ASFV proteins (Fig. 1E). Among the host-virus interactions, one between ASFV CP204L and the swine protein VPS39, a subunit of the HOPS complex, was notable in that it was identified with the highest abundance (log_10_ iBAQ [(intensity-based absolute quantitation]) (Data Set S1C). The HOPS complex plays a role in endosomal cargo trafficking by mediating endosomal maturation (29) and fusion of lysosomes with late endosomes, phagosomes, or autophagosomes (30, 31). Of the protein-protein interactions (PPIs) among viral proteins, the A137R protein was the most enriched ASFV protein interacting with CP204L (Data Set S1C). Unfortunately, we could not confirm the interaction between CP204L and A137R by an inverted pulldown (Fig. S1). Therefore, we focused on characterizing the interaction of CP204L with VPS39.

### CP204L interacts with the amino acid region from position 271 to 541 of the VPS39 subunit of the HOPS complex.

**FIG 2:**
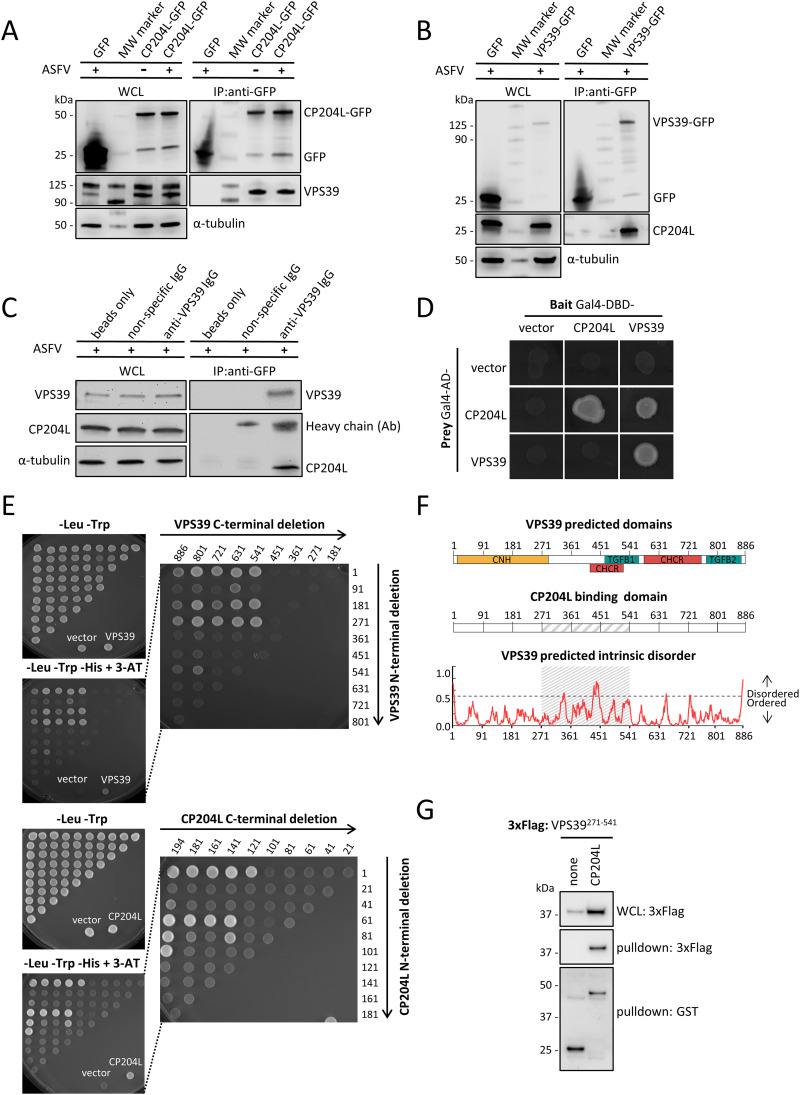
Interaction between ASFV CP204L and porcine VPS39. (A) Coimmunoprecipitation of GFP-tagged CP204L with endogenous VPS39. GFP was immunoprecipitated in lysates from mock-infected and virus-infected cells stably expressing CP204L-GFP or GFP alone. Representative immunoblots of whole-cell lysates (WCLs) and GFP immunoprecipitates (IPs) are shown. α-Tubulin was used as a loading control in WCLs. (B) Reverse coimmunoprecipitation of VPS39-GFP with CP204L in virus-infected cells. (C) WSL cells were infected with ASFV at an MOI of 2 for 24 h. Lysates were prepared and immunoprecipitated with rabbit anti-VPS39 antibody, a nonspecific rabbit IgG control, or beads only, followed by immunoblotting. (D) Interaction between CP204L and VPS39 was tested in a yeast two-hybrid assay. CP204L and VPS39 were expressed as fusion proteins with the DBD or AD domain of Gal4. The empty vector is shown as a negative control. (E) Yeast two-hybrid mapping of the CP204L-binding region in VPS39 and the VPS39-binding region in CP204L. Various combinations of VPS39 truncations and CP204L were cotransformed into the pPC86 vector. The transformants were spotted on control plates (−Leu −Trp) and selective plates (−Leu −Trp −His +3-AT [5 mM]). Cotransfection with interacting protein fragments was indicated by growth on the selective medium. Vertical and horizontal axes indicate the first and the last amino acid residues of each tested fragment, respectively. (F) (Top) Predicted domain organization of porcine VPS39. CNH, citron homology domain; CHCR, clathrin heavy-chain repeat; TGFB1 and TGFB2, transforming growth factor beta receptor-associated domains 1 and 2. (Middle) CP204L-binding region between amino acids 271 and 541 of VPS39. (Bottom) Degree of order predicted by IUPred2A. The cutoff was set to a 0.5 probability score. (G) HEK-293T cells were transfected with expression vectors encoding GST alone or fused to CP204L and tested for interaction with 3×FLAG-tagged VPS39^271–541^. WCLs were prepared at 48 h posttransfection (top), and copurifications of VPS39^271–541^ were assayed by pulldown using glutathione-Sepharose beads (middle and bottom).

We performed a coimmunoprecipitation (co-IP) experiment with anti-GFP agarose beads in the presence of GFP-tagged CP204L or GFP alone as a control. Endogenous VPS39 coprecipitated with CP204L-GFP in uninfected and infected cells, whereas no interaction was observed between VPS39 and GFP (Fig. 2A). Reverse co-IP experiments with GFP-tagged VPS39 in infected cells further confirmed the interaction between CP204L and VPS39 (Fig. 2B). Finally, we confirmed the interaction of endogenous VPS39 and CP204L in infected WSL cells (Fig. 2C). The IP assays were carried out with anti-VPS39 antibody or nonspecific IgG bound to protein G-conjugated magnetic beads. Beads alone were used as an additional control.

To determine whether the CP204L-VPS39 interaction is mutual, we applied a yeast two-hybrid (Y2H) assay. For this purpose, VPS39 and CP204L were fused to either the Gal4 DNA-binding domain (DBD) or the Gal4 activation domain (AD), and fusion proteins were expressed in yeast as bait or prey, respectively. Physical interaction between CP204L-AD and VPS39-DBD proteins was confirmed by the Y2H assay (Fig. 2D). Moreover, both proteins showed the ability to form homodimers using the Y2H system. To further map the region of VPS39 required for the interaction with CP204L, gap repair cloning was applied. Forward and reverse primers were designed for every 270 nucleotides along the VPS39 sequence (corresponding to 90 amino acids) and every 60 nucleotides along the CP204L sequence (corresponding to 20 amino acids). Fragments of VPS39 or CP204L were introduced into the Gal4-DBD vector and coexpressed in yeast cells grown on a selective medium with CP204L or VPS39 fused to Gal4-AD, respectively.

The results of this experiment indicated a 270-residue region encompassing amino acid positions 271 to 541 of VPS39 protein to be critical for the interaction with CP204L (Fig. 2E). We could also demonstrate that the middle region of CP204L is required for interaction with VPS39; however, the minimum interacting domain could not be specified. According to the InterPro (32) prediction, the CP204L binding region of VPS39 contains a clathrin heavy-chain repeat (CHCR) domain. It also overlaps with a citron homology (CNH) domain located at the N terminus and with a transforming growth factor beta receptor-associated domain 1 (Fig. 2F). Furthermore, an intrinsically disordered region is situated within the CP204L binding domain between amino acids 440 and 460 of VPS39, as predicted by IUPred2A (33). Finally, to validate the CP204L binding domain, glutathione *S*-transferase (GST)-tagged CP204L protein was coexpressed with 3×FLAG-tagged VPS39^271–541^ in HEK-293T cells, and total lysates were purified 48 h later with glutathione-Sepharose beads. In agreement with data obtained by Y2H, we confirmed that the 271–541 fragment of VPS39 is sufficient to bind CP204L (Fig. 2G).

### CP204L and VPS39 colocalize and aggregate at the virus factory.

We next performed colocalization analyses by confocal immunofluorescence microscopy. To improve the detection of the host protein, we used WSL cells stably expressing GFP-tagged VPS39.

**FIG 3:**
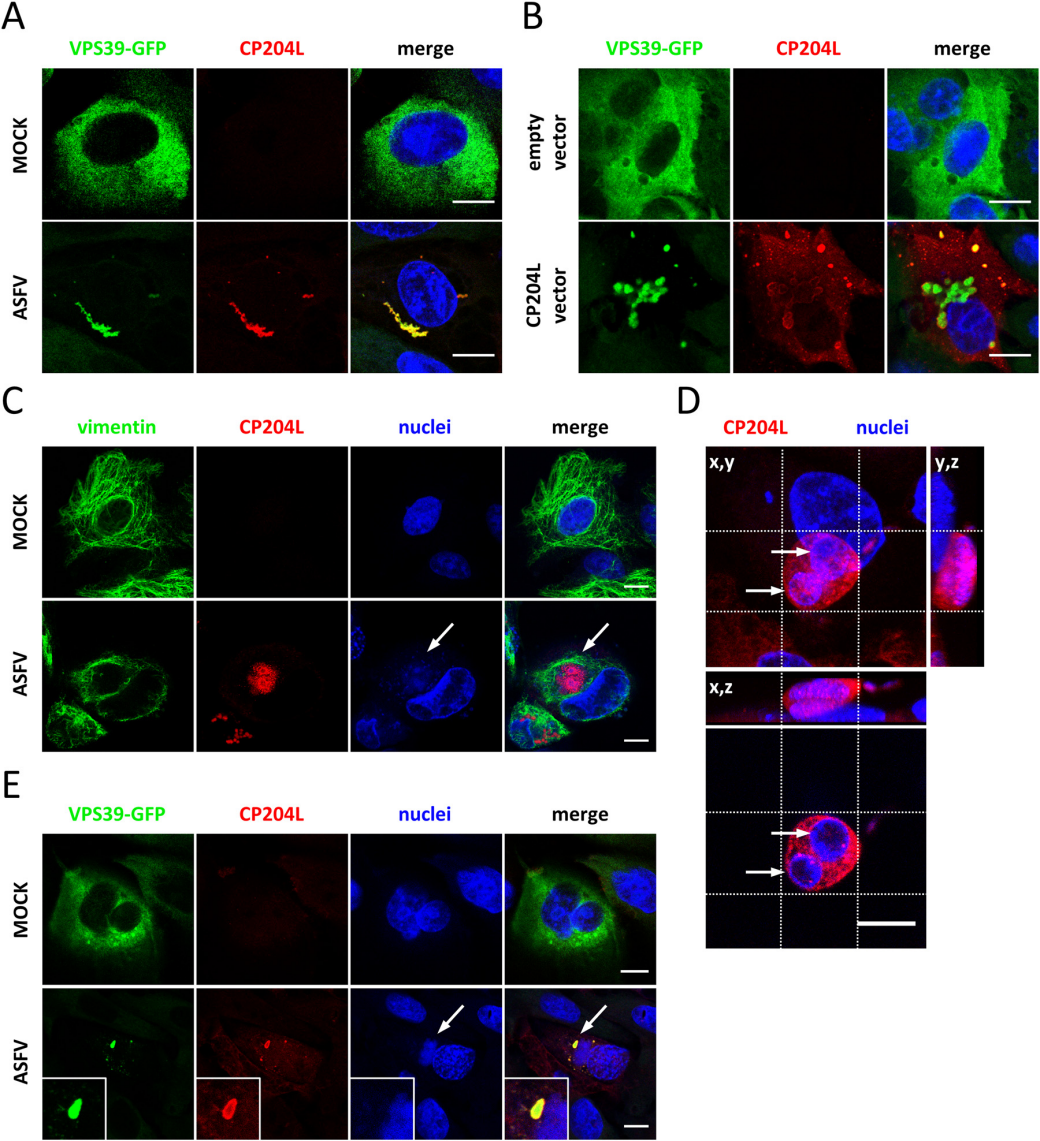
During infection, CP204L and VPS39 form aggregates and localize to the virus factory. Colocalization and aggregate formation between CP204L and VPS39 in WSL-VPS39-GFP cells (A) 24 h after ASFV infection and (B) CP204L plasmid transfection. Empty vector and mock-infected cells were used as negative controls. (C) Subcellular localization of CP204L in infected WSL cells (12 h after infection). Indirect immunofluorescence shows localization of CP204L (red) to virus replication sites (arrows) surrounded by a vimentin cage (green). (D) (Top) Representative image of a WSL cell infected with ASFV (24 h postinfection). The image shows a Z-stack projection (64 slices across 12.8 μm) of the cell nucleus and virus factories (blue) and the CP204L protein (red). The crosshairs were positioned to indicate virus DNA. (Bottom) Cross-section through the replication compartment and the CP204L accumulation at its periphery. (E) Protein VPS39 (green) and CP204L (red) colocalization at the periphery of the virus replication site in infected WSL-VPS39-GFP cells. Hoechst dye was used to stain cellular and virus DNA (blue). Arrows indicate virus factories. Bars, 10 μm.

Cells were mock or virus infected and stained with anti-CP204L antibody after 24 h. In the context of ASFV infection, both proteins colocalized in large cytoplasmic aggregates (Fig. 3A), whereas no VPS39 aggregates were detected in the absence of ASFV. A transfection experiment with a CP204L expression plasmid was performed to exclude the possibility of other virus proteins or infection-dependent mechanisms mediating the formation of VPS39-CP204L aggregates. This experiment confirmed that transiently expressed CP204L alone promotes VPS39 aggregation (Fig. 3B). Interestingly, the VPS39-CP204L complex formed large aggregates in the perinuclear region with smaller and more uniformly distributed granules.

**FIG 4:**
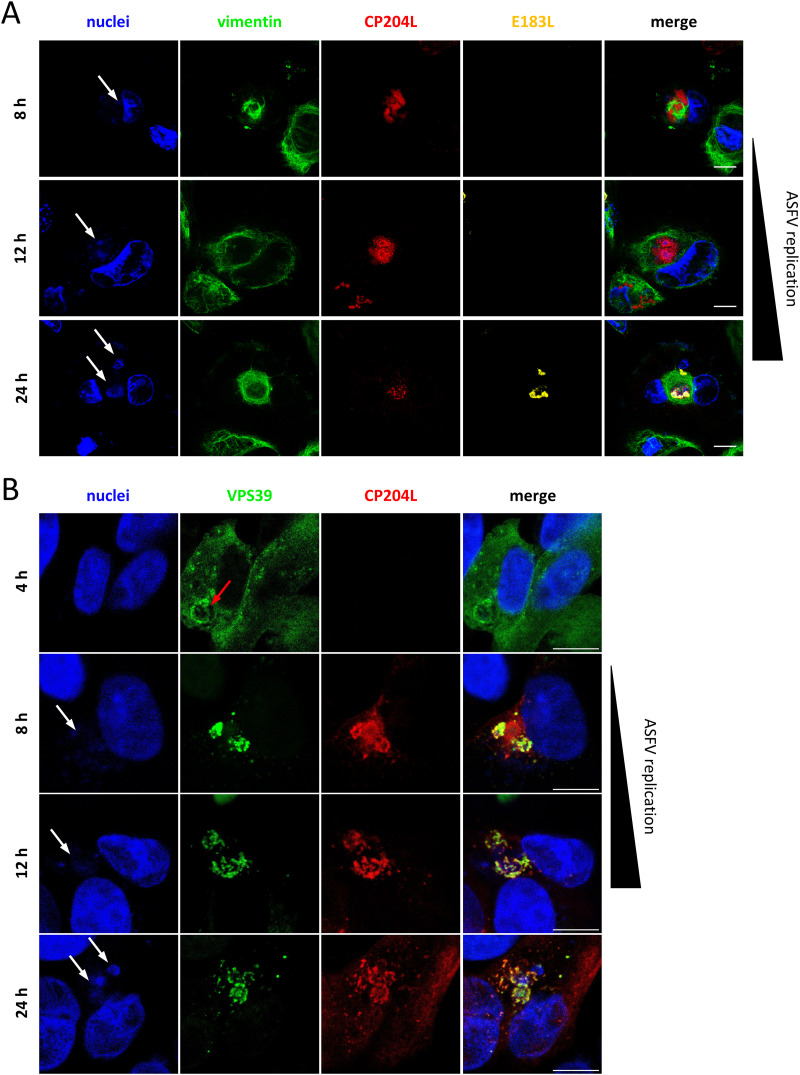
CP204L and VPS39 accumulate at the virus factory early during infection. (A) Virus factories in WSL cells infected with ASFV were monitored by immunofluorescence at 8, 12, and 24 h postinfection. Representative images show the accumulation of CP204L (red) in early VFs and dispersion from late VFs, which are marked by the presence of late virus protein E183L (P54) (yellow). Virus factories (white arrows) are labeled by DNA staining (blue) and staining of vimentin cages (green). Bars, 10 μm. (B) Colocalization of CP204L and VPS39 in ASFV-infected WSL-VPS39-GFP cells was monitored at 4, 8, 12, and 24 h postinfection. The aggregation of VPS39-GFP near the nucleus was observed 4 h postinfection (red arrow).

This observation led to the question of whether CP204L is targeted to a specific cellular structure in virus-infected cells in the absence of overexpressed VPS39. To address this question, WSL cells were infected with ASFV, and CP204L was visualized by indirect immunofluorescence 24 h postinfection. We noticed that CP204L exhibited distinct distribution patterns in infected cells. In addition to an even distribution in the cytoplasm, which has been described in previous studies (17, 24), it accumulated at the site of virus factories that are enclosed in characteristic vimentin cages (Fig. 3C). Z-stack sectioning and imaging of infected cells showed the accumulation of CP204L around the sites of virus DNA replication (Fig. 3D). As expected, we could also confirm that VPS39 aggregates together with CP204L at the periphery of the virus DNA replication sites (Fig. 3E). The localization of CP204L changes during infection, and its accumulations are significantly larger in early VFs than the late ones (Fig. 4A). VPS39 starts aggregating in the perinuclear area as early as 4 h postinfection. However, the colocalization with CP204L in the VF could be observed earliest at 8 h postinfection (Fig. 4B). Whereas VPS39 remains concentrated in the VF throughout the infection, some CP204L in the late stage of infection (after 24 h) showed cytoplasmic distribution.

### CP204L blocks VPS39 interaction with the HOPS complex and promotes lysosomal clustering.

**FIG 5:**
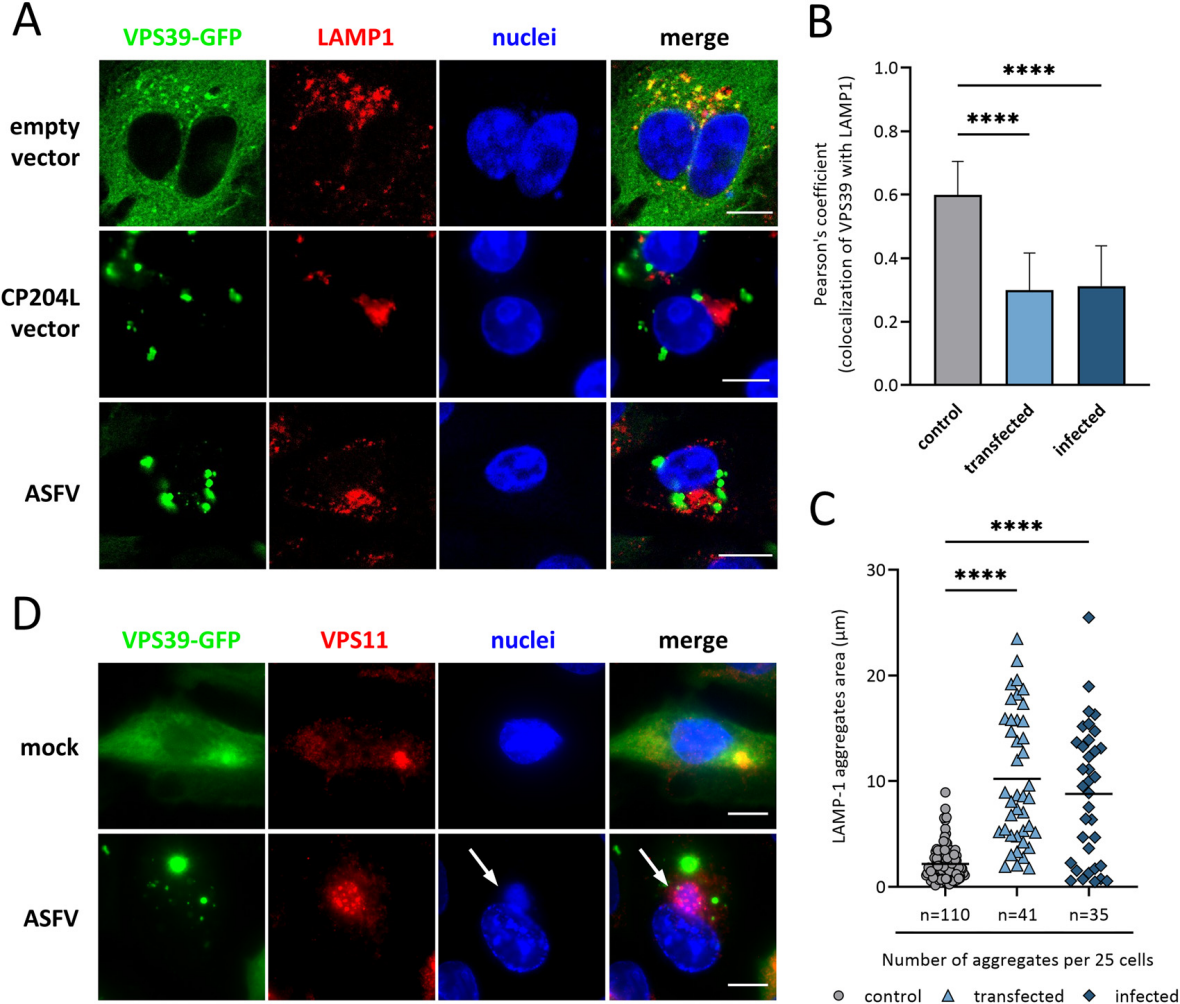
CP204L promotes lysosomal clustering and blocks VPS39 integration into the HOPS complex. (A) WSL cells stably expressing VPS39-GFP were analyzed by confocal fluorescence microscopy after transfection with a CP204L expression plasmid or infection with ASFV. Cells were stained for the lysosomal marker anti-LAMP1. (B) Quantification of the colocalization of VPS39 with lysosomes labeled by LAMP1. Pearson’s coefficient (mean ± standard error of the mean [SEM]) from 25 cells in each group. (C) Quantification of the number and area of lysosomal and late endosomal aggregates per cell (*n* = 25 cells in each group). ****, *P < *0.0001. (D) VPS11 staining shows a loss of colocalization between VPS39 and VPS11 in virus-infected cells. Arrows indicate virus factories. Bars, 10 μm.

Having established that both CP204L and VPS39 interact and aggregate at the virus factory, we sought to determine whether this interaction could impair VPS39 function in late endosomal trafficking (34). The WSL cells expressing VPS39-GFP were transfected with a vector for CP204L expression or infected with ASFV. Cells expressing an empty vector were used as a control. After 24 h, the colocalization of VPS39 with lysosome-associated membrane protein 1 (LAMP1) was examined by immunofluorescence microscopy. Control cells exhibited the characteristic cytoplasmic and juxtanuclear distribution of lysosomes, and LAMP1 puncta colocalized with VPS39 (Fig. 5A). In the cells expressing CP204L after transfection or after infection, VPS39 aggregates were largely separated from LAMP1-marked lysosomes, which concentrated in the area near the nucleus. When CP204L was present, colocalization between the LAMP1-marked lysosomes and VPS39 was significantly reduced (Fig. 5B). Similarly, CP204L expression altered the colocalization between VPS39 and Rab7-marked late endosomes (Fig. S2). Moreover, the expression of CP204L was accompanied by reduced numbers of LAMP1-positive puncta and a significant increase in the size of accumulated lysosomes, suggesting lysosomal enlargement through coalescence (Fig. 5C).

Finally, to test if the interaction with CP204L prevents the recruitment of VPS39 to the HOPS complex, we evaluated the colocalization of VPS39 with the subunit VPS11, which anchors VPS39 to the HOPS complex (35). Unlike in uninfected cells, no colocalization between VPS39 and VPS11 was observed in virus-infected cells (Fig. 5D). However, a clear colocalization between viral DNA (Hoechst staining) and VPS11 was detected, suggesting that VPS11 is targeted to the ASFV replication site independent of VPS39. Together, these observations indicate that CP204L inhibits the integration of VPS39 into the HOPS complex, thus interfering with VPS39 function to associate with endosomal and lysosomal membranes and leading to the clustering of lysosomes in the infected cells.

### ASFV replication and protein synthesis are delayed in cells lacking VPS39.

**FIG 6:**
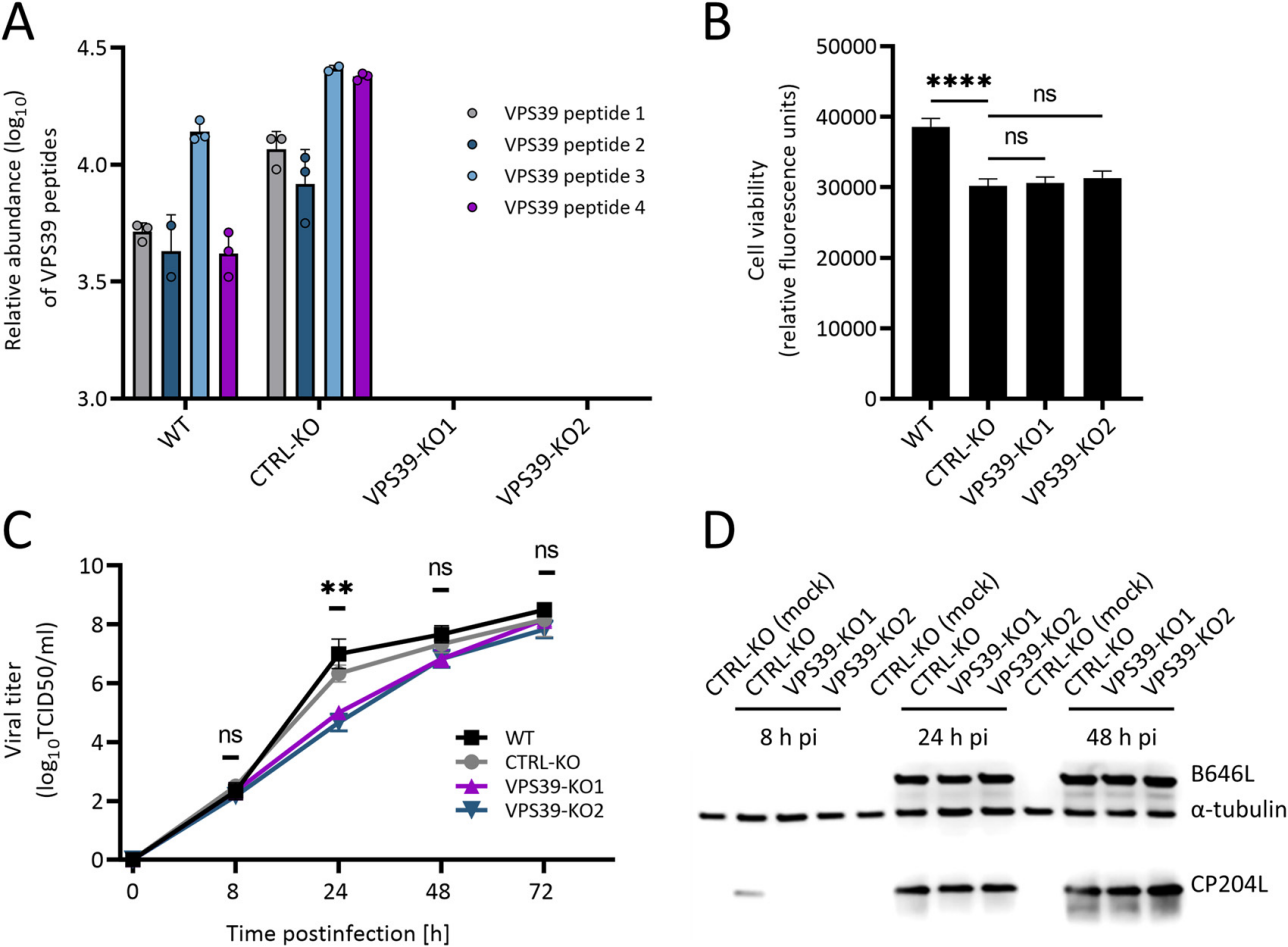
Absence of VPS39 delays ASFV growth and CP204L synthesis. (A) The absence of VPS39 in WSL KO cells was confirmed by mass spectrometry (Data Set S1D). VPS39 was identified based on four unique peptides in wild-type (WT) WSL cells and control knockout (CTRL-KO) cells. In VPS39-KO cells, no peptides of VPS39 were detected. The log_10_ relative abundance is presented for each peptide identified from three independent experiments. (B) Cell viability of WSL WT, CTRL-KO, and VPS39-KO cell lines was quantitated with a resazurin-based assay (PrestoBlue). ****, *P < *0.0001. (C) Growth curves of ASFV after infection of VPS39-KO and control cells at an MOI of 1 (*n* = 3 wells/cell line/time point). The culture medium was collected at the indicated times, and the yields of the cell-free virus were expressed as TCID_50_ per milliliter and were plotted as means of results from three independent replicates and standard deviations. **, *P < *0.01. (D) Expression of CP204L and B646L virus proteins in VPS39-KO and CTRL-KO cells. Mock-infected CTRL-KO cells were used as controls. The α-tubulin-specific monoclonal antibody was used as a loading control.

Next, we studied the role of the CP204L-VPS39 interaction in establishing the ASFV infection. CRISPR-Cas9 technology was used to generate a VPS39 knockout (KO) in the WSL cell line. The *VPS39* gene of the selected WSL cell clones exhibited a single nucleotide (A) insertion inducing a frameshift behind codon 202 (VPS39-KO1) and 101 (VPS39-KO2), leading to premature termination at positions 224 and 103, respectively. Both cell clones were homozygous with respect to these mutations and unable to express the CP204L binding domain localized between amino acids 271 and 541 of VPS39. The absence of VPS39 was confirmed in the VPS39-KO cells by mass spectrometry, where no peptides corresponding to the VPS39 protein were detected in VPS39-KO1 and VPS39-KO2 (Fig. 6A; Data Set S1D). Importantly, VPS39 deficiency in WSL cells did not reduce the cell viability compared to empty vector control cells (CTRL-KO) (Fig. 6B). The moderately reduced viability of all knockout cells, compared to wild-type cells, could be an unspecific deleterious effect of Cas9 nuclease, which was also abundantly expressed in the CTRL-KO cells.

Next, KO cells were infected with ASFV at a multiplicity of infection (MOI) of 1 and incubated for 8, 24, or 48 h. Progeny virus titers were reduced by 1.5-log_10_ in VPS39-KO compared to control cell supernatant 24 h after infection (Fig. 6C). However, final ASFV titers in VPS39-KO cells at 72 h were not different from those in WT or CTRL-KO cells, suggesting that ASFV can replicate in the absence of VPS39, but with delayed kinetics. Additionally, the lysates of KO cells infected with ASFV for 8, 24, or 48 h were harvested for Western blotting to examine the expression of early (CP204L) and late (B646L) virus proteins. While the expression of capsid protein B646L (P72) was similar in CTRL-KO and VPS39-KO cells, CP204L expression appeared to be delayed in cells lacking VPS39 (Fig. 6D). This observation raised the question of whether the synthesis of other early virus proteins expressed before the onset of virus DNA replication is also affected by the lack of VPS39.

**FIG 7:**
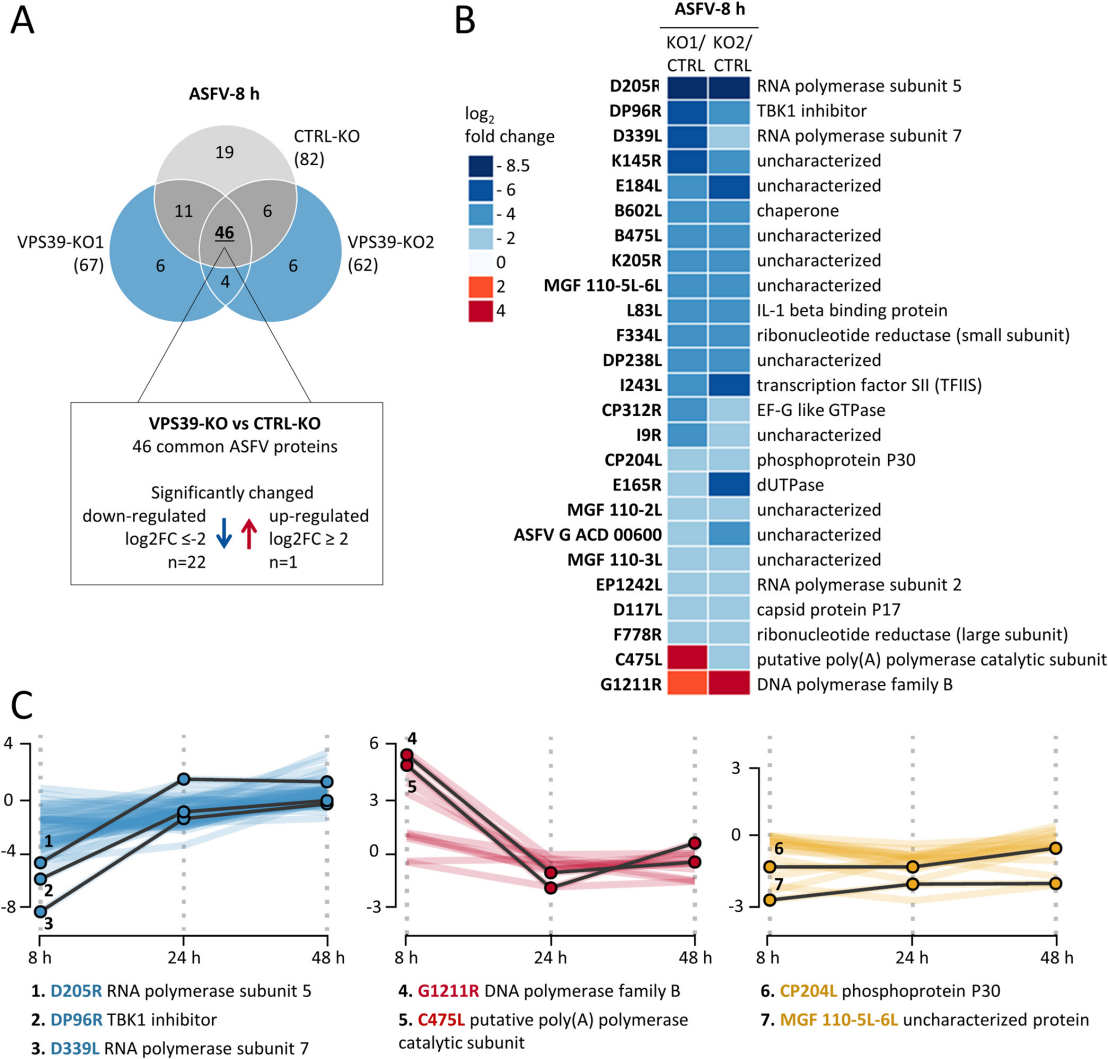
Loss of VPS39 affects the synthesis of ASFV proteins during the early phase of infection. (A) Overview of identified ASFV proteins 8 h after infection in CTRL-KO and VPS39-KO cells. The number of proteins exhibiting reduced or increased amounts in VPS39-KO cells was determined. (B) Heat map of viral proteins identified at 8 h depicts changes in protein expression levels between CTRL-KO and VPS39-KO cells. (C) Line plots showing fold changes in ASFV proteins expressed in VPS39-KO cells compared to controls. Three clusters with similar profiles of protein were distinguished: downregulated (or unchanged) at 8 h and increasing over time (blue); upregulated (or unchanged) at 8 h and decreasing over time (red); and continuously downregulated during infection in VPS39-KO cells (yellow). Selected ASFV protein profiles are depicted with solid black lines. Mass-spectrometric data can be found in Data Set S1D.

To answer this question, we first analyzed the changes in virus protein levels in CTRL-KO and VPS39-KO cells 8 h after ASFV infection by quantitative MS. Among 46 virus proteins identified in all three cell lines, 22 were significantly downregulated in cells lacking VPS39 (Fig. 7A). In particular, we observed a decrease in levels of virus proteins implicated in RNA transcription (i.e., ASFV RNA polymerase subunits: D205R, EP1242L, I243L, and D339L) and DNA replication (i.e., ribonucleotide reductase: F334L, F778R, and dUTPase E165R) (Fig. 7B). We further analyzed the dynamics of viral protein synthesis in VPS39-KO cells across the indicated time points (Fig. 7C; Data Set S1D). As expected based on ASFV growth kinetics, the most profound changes were observed at 8 h after infection, whereas at later stages of infection, the levels of viral proteins in VPS39-KO cells stabilized and were comparable with those observed in CTRL-KO cells. These results suggest that although a loss of VPS39 markedly delays the synthesis of CP204L and other early proteins, VPS39 is not essential, and ASFV replication can proceed in its absence.

## DISCUSSION

Protein interaction networks yield critical insights into the virus-host interrelationships and mechanisms of viral protein function. In this study, the protein interaction network derived from AP-MS data provided new information about the molecular hijacking strategies of ASFV and the role of virus protein CP204L. We found it particularly interesting that CP204L establishes a rich network of interactions with host vesicle trafficking proteins.

Vesicular trafficking is a directed cellular process of transporting cargo to target locations inside or outside the cell. It is, therefore, not surprising that viruses usurp this pathway to achieve efficient transport within the host cell. However, the role of host trafficking vesicles extends beyond virus movement, as the formation of virus-modified endosomal membranes supports viral replication, reported especially for RNA viruses (reviewed in reference 36). It has been proposed that the cholesterol-rich endosomes can provide lipids for ASFV nucleic acid replication and virus assembly, structural scaffolding for viral factories, and protection from antiviral host responses (19, 37). However, the precise contributions of cellular factors and mechanisms underlying endosomal recruitment and accumulation at the ASFV replication site remain largely enigmatic. Our study sheds new light on the mechanism of endosomal membrane redistribution during ASFV infection by identifying novel viral and cellular proteins involved in this process.

Protein A137R was the most enriched among all viral proteins detected by AP-MS analysis. However, the reverse pulldown failed to confirm the interaction between CP204L and A137R (Fig. S1). Sun et al. (38) found that A137R inhibits type I interferon production by interacting with TANK-binding kinase 1 (TBK1) and promotes its lysosomal degradation by a yet-unknown mechanism. As both proteins, A137R and CP204L, are involved in the endocytic pathway, they may interact directly or indirectly by sharing interaction partners. The ectopic expression of A137R-GFP fusion protein or structural changes induced by the protein fusion may interfere with binding to certain ligands, e.g., CP204L. Further experiments are necessary to characterize the nature of CP204L and A137R interaction.

The important initial observation of this study was that the CP204L-host interactome is significantly enriched in proteins associated with vesicle transport and mitochondria (Fig. 1D). Within these two functional groups, VPS39, a subunit of the vacuolar HOPS tethering complex, and TOMM22, a subunit of the translocase of the outer membrane (TOM) mitochondrial complex, were the most abundant host interactors of ASFV CP204L (Fig. 1E). Interestingly, the interaction between the TOM complex and VPS39 was previously reported to be essential for the formation of membrane contact sites (MCSs) between cellular vesicles (e.g., endosomes and lysosomes) and mitochondria (39, 40). Only recently have MCSs emerged as important host cell structures that enable viruses to reorganize the cellular membranes and channel cell lipids to the growing replication centers (41, 42). Therefore, it is tempting to speculate that the interaction between VPS39, TOMM22, and CP204L could play a role in establishing MCSs in ASFV-infected cells. However, to focus specifically on the mechanism by which ASFV exploits the endocytic pathway, this study solely investigated the interaction between VPS39 and CP204L.

Interestingly, VPS39 was not identified as a CP204L interactor in the Y2H screen performed by Chen et al. (27), and none of the 7 interactors identified in their study appeared as a specific interactor in our data set. However, it is unclear whether VPS39 was present in the cDNA library derived from porcine alveolar macrophages and used by Chen et al. (27). Nevertheless, a similar conclusion was reached by Chen et al. and us, i.e., that CP204L plays a role in vesicle trafficking by interacting with proteins involved in the endocytic pathway.

The VPS39 subunit was identified as the most confident interactor of CP204L by the PPI screen (Fig. 1E). Nonetheless, other subunits of the HOPS complex, such as VPS11, VPS16, and VPS18, but not VPS41 and VPS33, were also detected. For now, we ascribe this to the indirect binding of those proteins to the complex formed by the bait and to their oligomerization properties (43) rather than to a specific and direct binding to the CP204L. Our data show that CP204L individually targets the VPS39 subunit, blocking its attachment to the HOPS complex via VPS11. However, we cannot exclude the possibility that CP204L could also sequester other HOPS complex subunits. We showed that CP204L binds VPS39, and the binding site could be mapped to amino acids 271 and 541 of VPS39 (Fig. 2). We could also confirm the formation of homo-oligomer for both proteins, CP204L (44) and VPS39 (45). Oligomerized CP204L is present in infected cells mostly as hexamers, and the existing dimers are suggested to serve as assembly units for the final oligomerization (44). While oligomerization is a common feature of many viral proteins, the specific mechanism and role of the CP204L oligomerization are not clear. In this context, it is interesting that, e.g., Ebola virus VP40 (46), influenza virus matrix protein M1 (47), and Dengue virus NS1 (48) undergo oligomerization when binding to cellular membranes. The formation of oligomers and the presence of intrinsically disordered regions in viral proteins enhance diverse interactions with host proteins, resulting in protein multifunctionality (49).

Previously, Hernaez et al. (26) showed that a small portion of CP204L is present in the nucleus, interacting with heterogeneous nuclear ribonucleoprotein K (hnRNP-K) and causing its retention in the nucleus. To date, CP204L has been described as a predominantly cytoplasmic ASFV protein. In this study, we show that CP204L localizes to VFs, specific intracellular sites of virus replication (Fig. 3). The localization of CP204L varies during infection, and its accumulation at the VFs occurs at early times, before ASFV late protein synthesis (Fig. 4). We suggest that the difference in CP204L protein sequence between ASFV isolates may modulate its targeting to the VF. Previously, cytoplasmic localization of CP204L was reported in Vero cells infected with the BA71V isolate of ASFV. It has been reported that the protein CP204L of BA71V has a 10-amino-acid extension at the C terminus (50), which is not present in CP204L of the ASFV isolate used in this work. Moreover, the polyclonal antibody used in our study can likely recognize different epitopes than the monoclonal antibodies used previously. Thus, different forms of CP204L, such as monomer, dimer, hexamer, etc., could be localized by our antibody. Furthermore, we also detected a considerable proportion of CP204L dispersed throughout the cytoplasm during the late phase of infection.

Interestingly, immunofluorescence analysis revealed that CP204L localized within VFs is clearly separated from viral DNA, suggesting its possible role in viral protein transcription and translation rather than virus replication. Also, the fact that a large number of ASFV proteins were found to coimmunoprecipitate with CP204L (Fig. 1E) supports its possible role in the process of viral protein synthesis. On the other hand, it is also possible that CP204L interacts with proteins of membrane-bound organelles such as mitochondria, the endoplasmic reticulum, or endosomes/lysosomes, which are all recruited to the area next to the virus DNA replication site.

Recently, Miao et al. (51) and Zhang et al. (52) reported interaction of SARS-CoV-2 protein ORF3a with the HOPS component VPS39. In this case, late-endosome-localized ORF3a sequesters VPS39 and, consequently, inhibits autophagy by blocking the fusion of autophagosomes with lysosomes. Our results indicate that the mechanism by which ASFV protein CP204L interacts with VPS39 differs from the one used by severe acute respiratory syndrome coronavirus 2 (SARS-CoV-2). First, CP204L prevents VPS39 from binding to endosomal/lysosomal membranes (Fig. 5A). Second, CP204L expression inhibits VPS39 binding to VPS11 and its integration into the HOPS complex (Fig. 5D), while SARS-CoV-2 ORF3a expression does not affect the formation of the VPS39-containing HOPS complex (51). Third, the expression of CP204L leads to clustering of lysosomes (Fig. 5C) and accumulation of CP204L-VPS39 aggregates near ASFV VFs (Fig. 3E).

CP204L interacts with VPS39 within its clathrin heavy-chain repeat (CHCR), which is required for association with endosomal membranes via binding to Rab7-interacting lysosomal protein (RILP), and homooligomerization of VPS39 (53). Rab7 was already shown to be essential for ASFV replication (13). Therefore, the significant decrease in VPS39-lysosome colocalization during ASFV infection can be explained by a simple competition model wherein highly abundant CP204L competes with host RILP for binding to VPS39 in the cytosol. The loss of the ability of VPS39 to bind VPS11 and, consequently, to form the HOPS complex in the presence of CP204L could be explained either by direct competition of CP204L and VPS11 for the same binding site on VPS39 or by an allosteric effect of the CP204L-VPS39 interaction on the VPS39 binding site for VPS11.

CP204L is essential for the virus, and the reduction of protein levels results in a strong suppression of viral replication (21). Although virus replication (Fig. 6) and protein synthesis (Fig. 7) were reduced in VPS39-deficient cells, especially early during infection, this effect did not correlate with the 4-order-of-magnitude reduction observed with CP204L inhibition. This observation suggests that CP204L engages in several distinct functions important for virus replication, one of which involves the interaction with VPS39.

Nevertheless, we propose that the hijacking of VPS39 by CP204L may have two possible functions during ASFV infection. First, by inhibiting VPS39 association with endosomes and lysosomes, CP204L may impair homotypic and heterotypic fusion of vesicles, thus protecting endocytosed viruses from degradation. Second, by CP204L-induced dissociation of VPS39 from the HOPS complex and endosomal membranes, VPS39 gains the ability to engage in membrane contact sites formation (39), playing a role in the biogenesis of ASFV VFs. In this case, the loss of VPS39 could affect the ASFV membrane synthesis and virus assembly, thus leading to the observed delay in virus replication.

In summary, based on our results and previous studies, we conclude that CP204L is a multifunctional protein that interacts with the VPS39 subunit of the HOPS complex, is involved in endosomal trafficking, and orchestrates late endosome and lysosome clustering. CP204L exists in multiple oligomeric forms, undergoes phosphorylation, and localizes to the cytoplasm, nucleus, and virus factory. Moreover, we showed that VPS39 plays a role in the early phase of ASFV replication and protein synthesis, but it is not essential for ASFV infection progression in wild boar cells.

## MATERIALS AND METHODS

### Cells and virus.

The wild boar lung-derived cells (referred to as WSL cells here) (54), supplied by Friedrich-Loeffler-Institut Biobank (catalog number CCLV-RIE 0379), were maintained at 37°C with 5% CO_2_ in Iscove’s modified Dulbecco’s medium (IMDM) mixed with Ham’s F-12 nutrient mix (1:1 [vol/vol]) supplemented with 10% fetal bovine serum (FBS). ASFV (Armenia/07 isolate) was adapted by serial passaging to more efficient replication in WSL cells. Passage 20 stocks were generated as described previously (21).

### ASFV *in vitro* infection.

All experiments with ASFV were performed in a biocontainment facility fulfilling the safety requirements for ASF laboratories and animal facilities (Commission Decision 2003/422/EC, chapter VIII). For infection experiments, WSL cell monolayers were inoculated with ASFV stock dilutions at an MOI of 2 PFU/cell, and supernatants collected from uninfected cells were used for the mock-infected controls. After inoculation, cells were centrifuged for 1 h at 600 × *g* and 37°C. Next, cells were washed three times with phosphate-buffered saline (PBS), replenished with a medium containing 5% FBS, and incubated at 37°C with 5% CO_2_. Supernatants were harvested at appropriate times, and progeny virus titers were determined as 50% tissue culture infective doses (TCID_50_) per milliliter (55) on WSL cells.

### DNA transfection.

WSL cells were transiently transfected with a CP204L, a VPS39, or an empty vector using K2 multiplier and K2 transfection reagent (Biontex) following the manufacturer’s instructions. Stable cell lines were generated by transient DNA transfection of WSL cells with plasmids encoding GFP, CP204L-GFP, or VPS39-GFP. Three days after transfection, cells were trypsinized, seeded into 96-well plates, and maintained in a medium containing 500 μg/mL G418 (Corning). Single resistant and fluorescent cell clones detected after 2 to 3 weeks were further propagated and validated for expression of GFP fusion protein by immunoblotting.

### Plasmids constructs.

**(i) Control plasmids (GFP and empty vector).** pGFP-N1 plasmid (Clontech; GenBank accession no. U55762) was used for GFP expression. To obtain a matching control plasmid for transfection experiments, a 741-bp BamHI/NotI fragment containing the open reading frame (ORF) encoding the enhanced GFP was deleted from pGFP-N1 (Clontech; GenBank accession no. U55762), resulting in pΔGFP-N1 after Klenow treatment and ligation.

**(ii) CP204L-GFP plasmid.** For the generation of CP204L-GFP plasmid, the codon-adapted viral frame (ORF) CP204L of ASFV Georgia 2007 (GenBank accession no. FR682468) (28) was amplified from plasmid pUC-BaKJCAG-CP204Lsyn (56) by PCR with primers pCAG-F3 and ASFVp30CDS-R (Table S1) using KOD Xtreme Hot Start DNA polymerase (Sigma/Merck). After digestion of the PCR product with BamHI and EcoRI, the isolated 617-bp fragment was inserted into the correspondingly digested and dephosphorylated reporter gene expression vector pGFP-N1 (Clontech; GenBank accession no. U55762). In the resulting plasmid, CP204L-GFP, the synthetic CP204L ORF was under the control of the human cytomegalovirus (HCMV) immediate early promoter/enhancer complex and 3′-terminally fused to the coding sequence of an enhanced GFP.

**(iii) VPS39-GFP plasmid.** For overexpression of VPS39 and GFP fusion proteins with VPS39 in transfected eukaryotic cells, the predicted gene product of Sus scrofa (isoform X3; GenBank accession no. XP_013848582) was back-translated in line with porcine codon preferences. The custom-made (GeneArt, Thermo Fisher Scientific) synthetic ORF was flanked by a 5′-terminal Kozak sequence (CCACC) and restriction sites for convenient recloning. For fusion of VPS39 to the N terminus of GFP, a 2,674-bp EcoRI/BamHI fragment was inserted into correspondingly digested pGFP-N1. The obtained precursor plasmid was digested with AhdI, treated with Klenow polymerase, and religated. This treatment caused a frameshift immediately upstream of the stop codon of VPS39, leading to in-frame fusion with the downstream GFP ORF in pVPS39porc-GFP.

**(iv) A137R-GFP plasmid.** A137R of ASFV Georgia 2007 (GenBank accession no. FR682468) (28) was generated by gene synthesis (Twist Bioscience) and shuttled via Gateway technology (Invitrogen) into the pcDNA6.2/N-emGFP-DEST plasmid backbone (Invitrogen) to generate A137R-GFP plasmid. All plasmid constructs were verified by DNA sequencing.

### Affinity purification and mass spectrometry.

**(i) Affinity purification.** A total of 5 × 10^6^ WSL cells (stably expressing GFP, CP204L-GFP, or VPS39-GFP) were mock infected or infected with cell culture-adapted ASFV Armenia 2008 at an MOI of 2 PFU/cell. After 24 h, cells were washed three times with PBS and lysed on ice in 1 mL cold immunoprecipitation (IP) buffer (50 mM Tris-HCl [pH 7.4], 150 mM NaCl, and 1 mM MgCl_2_ supplemented with Benzonase [25 U/mL; Sigma-Aldrich no. E8263], 0.5% Nonidet P40 substitute [NP-40; Sigma-Aldrich no. I8896], and cOmplete mini EDTA-free protease inhibitor cocktail [Roche, no. 04693159001]). Cells were lysed by sonication three times for 30 s each at 80% amplitude (Branson Digital Sonifier 450) and incubated on a tube rotator for 30 min at 4°C and another 30 min on a thermomixer at 37°C with constant shaking at 900 rpm. Lysates were cleared by centrifugation at 13,000 × *g* and 4°C for 15 min. Fifty microliters of each lysate was removed for immunoblotting (whole-cell lysate fraction). GFP-trap agarose beads (50 μL; Chromotek) were washed twice in IP buffer and incubated with the remaining lysate for 1 h with constant rotation at 4°C. Beads were washed twice with 1 mL of IP buffer containing 0.05% NP-40 followed by two washes in detergent-free IP buffer. Ten microliters of bead slurry was removed for immunoblotting (IP fraction). The remaining 40 μL beads prepared in four independent biological replicates for CP204L-GFP without infection, CP204L-GFP with ASFV infection, and GFP with ASFV infection and in three independent biological replicates for GFP without infection were kept for mass-spectrometric analysis.

**(ii) Sample preparation for mass spectrometry.** After AP, beads were suspended in 300 μL freshly made UA buffer (8 M urea, 100 mM Tris-HCl [pH 8]), loaded onto 10-kDa filter units (Sartorius), and centrifuged at 12,000 × *g* at 20°C for 30 min. Filter-aided sample preparation (FASP) trypsin digestion was performed as described previously (57). Beads were trypsinized to digest the baits and the interacting proteins in 100 μL of digest buffer (1 M urea, 50 mM Tris-HCl [pH 7.5], and 5 μg/mL trypsin [Promega]). Digestion was performed overnight at 37°C with shaking. The next day, the flowthrough containing peptides was collected, and the samples were inactivated for 10 min at 95°C. Peptides were acidified with formic acid (1% final concentration), desalted using C_18_ 100-μL tips (Thermo Scientific) according to the manufacturer’s instructions, dried by vacuum centrifugation, and reconstituted in 20 μL of 0.1% formic acid prior to mass spectrometry.

**(iii) Protein identification by LC-MS/MS.** Digested peptide mixtures were analyzed by liquid chromatography-tandem mass spectrometry (LC-MS/MS) on a timsTOF Pro (Bruker Daltonics), which was coupled online to a nanoElute nanoflow liquid chromatography system (Bruker Daltonics) via a CaptiveSpray nano-electrospray ion source. Peptides (corresponding to 100 ng) were separated on a reverse-phase C_18_ column (25 cm by 75 μm; inside diameter, 1.6 μm; IonOpticks) with a binary buffer system of buffer A (water with 0.1% formic acid [vol/vol]) and buffer B (acetonitrile with 0.1% formic acid [vol/vol]). Peptides were separated by running a gradient of 2 to 95% mobile phase B over 115 min (2% to 16% solvent B (0 to 60 min), 15 to 24% solvent B (60 to 90 min), 24% to 34% solvent B (90 to 105 min), 35 to 95% solvent B (105 to 107 min), and 95% solvent B (107 to 115 min) at a constant flow rate of 400 nL/min. The column temperature was controlled at 40°C. MS analysis of eluting peptides was performed in ddaPASEF mode (1.1-s cycle time) as recommended by the manufacturer.

**(iv) MS data analysis.** Mass spectrometry raw files were processed with MaxQuant (v.2.0.2.0) (58). A peptide search was performed against an ENSEMBL (59) Sus scrofa proteome database (v.11.1.2021-11-10) and an NCBI ASFV Georgia (v.FR682468.2) proteome database. The following modifications were included in the search parameters: trypsin digestion with a maximum of 2 missed cleavages, carbamidomethylation of cysteine as a fixed modification, and protein N-terminal acetylation and oxidation of methionine as variable modifications. Mass error tolerance was set to 20 ppm for the full scan (MS1) and 40 ppm for MS/MS (MS2) spectra. The false discovery rate (FDR) on the peptide and protein levels was set to 0.01, the minimum peptide length was seven amino acids, and the match-between-runs option was used with a 0.7-min match window and 20-min alignment time. The output files were analyzed using Perseus software (v.2.0.3.0) (60). Proteins identified only by modified peptides, reverse hits, and contaminants were filtered out. A protein was considered identified if at least 2 unique peptides of this protein were found in 50% of the replicates. Further filtering of proteins specifically binding to CP204L protein was achieved by comparing CP204L protein pulldown assays with the negative GFP control. iBAQ (intensity-based absolute quantitation) protein quantitation data were log_2_ transformed and filtered to contain a minimum of two valid values in at least one experiment. Missing values were imputed on the total matrix with default settings (width, 0.3; downshift, 1.8). Potential interactors were considered CP204L specific if they were identified only in CP204L pulldown assays or if the log_2_ fold change (between control and CP204L) was greater than 2 and the *q* value of a two-sided *t* test was <0.01.

### GO overrepresentation and network analysis.

The data sets obtained for the CP204L interactome in mock-infected and ASFV-infected cells were tested for enrichment of Gene Ontology (GO) biological process terms (61). Porcine genes were assigned to their corresponding human orthologues using the R package gprofiler2 (v.0.2.1) (62). The overrepresentation analysis was performed using the enricher function of the clusterProfiler (v.4.2.2) (63) package in R with default parameters. Significant GO terms (adjusted *P* value < 0.01) were identified and further clustered based on their semantic similarity using the R package rrvgo (v.1.6.0) (64). Selected preys were manually curated, and the network diagram was plotted using Cytoscape (v.3.7.2) (65).

### Antibodies.

Rabbit antisera specific for ASFV CP204L, B646L, E183L (56), and A137R (our unpublished data) proteins were used at dilutions of 1:20,000 for immunoblotting. The primary antibodies used for immunoblotting included rabbit anti-GFP (Chromotek), rabbit anti-VPS39 (PA5-21104; Thermo Fisher), mouse anti-tubulin (B-5-1-2; Sigma-Aldrich), and mouse anti-GAPDH (glyceraldehyde-3-phosphate dehydrogenase; MCA4739; Bio-Rad). The secondary antibodies used were peroxidase-conjugated goat anti-mouse and anti-rabbit IgG (Jackson ImmunoResearch). The additional primary antibodies used for immunofluorescence were mouse anti-vimentin (MA1-06908; Thermo Fisher), rabbit anti-Rab7 (PA5-52369; Thermo Fisher), mouse anti-LAMP-1 (MCA2315GA; Bio-Rad), and rabbit anti-VPS11 (PA5-21854; Thermo Scientific). The secondary antibodies were Alexa Fluor 647-conjugated goat anti-rabbit IgG (H+L) and goat anti-mouse IgG (H+L) (Invitrogen).

### Immunoprecipitation.

For immunoprecipitation of endogenous proteins, 4 μg of purified rabbit anti-VPS39 IgG antibody (PA5-21104; Thermo Fisher) or normal rabbit IgG (negative control) (2729; Cell Signaling) was conjugated to 50 μL Dynabeads protein G (Thermo Fisher) for 30 min at room temperature, followed by washing twice in IP buffer. Antibody-conjugated beads, or beads alone, were incubated with 500 μL whole-cell lysates of infected WSL (1 × 10^7^ cells) in IP buffer for 1 h at room temperature. Beads were washed three times in IP buffer and eluted with SDS-PAGE loading dye at 95°C for 5 min. Immunoprecipitation of ASFV CP204L was examined by SDS-PAGE, followed by immunoblotting.

### Immunoblotting.

Beads and whole-cell lysates were boiled in IP buffer, resolved on SDS-PAGE gels (4 to 20% Mini-Protean TGX gels (Bio-Rad) (66), and transferred to the nitrocellulose membrane by semidry transfer (Trans-Blot Turbo; Bio-Rad Laboratories) (67). All membranes were blocked in 5% milk powder in Tris-buffered saline with 0.25% Tween 20 (TBST) and probed overnight with the indicated primary antibodies using appropriate dilutions. This was followed by three 10-min washes in TBST and by incubation with peroxidase-conjugated secondary antibodies diluted in TBST. After 1 h, membranes were washed as described above, and protein bands were detected using the Clarity Western enhanced chemiluminescence (ECL) substrate (Bio-Rad), imaged on a C-DiGit blot scanner (LI-COR), and analyzed with Image Studio software (v.5.2).

### Immunofluorescence.

Coverslips were fixed with 3.7% formaldehyde in PBS for 60 min at room temperature, washed 3 times for 10 min with PBS, permeabilized with 0.01% Triton X-100 in PBS for 15 min, and then blocked with PBS containing 10% FBS for 1 h. Coverslips were incubated with the primary antibody for 1 h at 37°C and then with the secondary antibody for 1 h at 37°C. Nuclei were stained for 15 min with 1 μg/mL Hoechst 33258 (Sigma-Aldrich) in PBS. After each step, the cells were repeatedly washed with PBS. Coverslips were then mounted on glass slides using ProLong Glass Antifade Mountant (Invitrogen). Single-slice images were acquired on a Leica DMI6000 TCS SP5 confocal laser scanning microscope (63× objective) and were processed with ImageJ software (v.1.52a) (68). For colocalization analysis, Pearson’s coefficient (ImageJ Plugin JaCoP [69]) was used to quantify the degree of overlap between the stainings indicated in the confocal images.

### Yeast two-hybrid analysis of the protein interaction.

To identify the CP204L binding site, we used the yeast two-hybrid system. First, forward and reverse PCR primers were designed along the VPS39 sequence every 270 and 240 nucleotides. These primer sequences were fused to specific tails, allowing recombination in the Gal4-BD pDEST32 vector. The sequences of the specific tails were 5′-GAAGAGGTAGTAACAAAGGTCAAAGACAGTTGACTGTATCGTCGAGG-3′ and 5′-CCGCGGTGGCGGCCGTTACTTACTTAGAGCTCGACGTCTTACTTA-3′. Matrix combinations of forward and reverse primers were used to amplify VPS39 fragments by PCR. As previously described (70), 10 ng of linearized pDEST32 empty vector was cotransformed with 3 μL of PCR product to achieve recombinational cloning by gap repair in the Y2H Gold yeast strain (Clontech) expressing AD-fused CP204L (pPC86 vector). Interactions between VPS39 fragments and CP204L were tested by plating yeast cells on a selective medium lacking leucine, tryptophan, and histidine and supplemented with 5 mM 3-amino-1,2,4-triazole (3-AT).

### GST pulldown experiments.

HEK-293T cells were dispensed into each well of a 6-well plate (2 × 10^6^ cells) and 24 h later were transfected (JetPRIME; Polyplus) with 600 ng of pCI-neo-3xFLAG vector encoding porcine VPS39^271–541^ with pDEST 27 (Invitrogen) expressing either GST alone (800 ng) or GST-CP204L (400 ng). Two days posttransfection, cells were collected in PBS and then incubated on ice in lysis buffer (20 mM morpholinepropanesulfonic acid [MOPS]-KOH [pH 7.4], 120 mM KCl, 0.5% Igepal, 2 mM β-mercaptoethanol, supplemented with complete protease inhibitor cocktail [Roche]) for 20 min. Cell lysates were clarified by centrifugation at 14,000 × *g* for 30 min. For pulldown analysis, protein extracts were incubated for 2 h at 4°C on a spinning wheel with 30 μL of glutathione-Sepharose beads (Amersham Biosciences). Beads were then washed 3 times for 5 min with ice-cold lysis buffer and on a spinning wheel, and proteins were recovered by boiling in denaturing loading buffer (Invitrogen).

Purified complexes and protein extracts were boiled at 95°C for 5 min and resolved by SDS-polyacrylamide gel electrophoresis (SDS-PAGE) on 4 to 12% NuPAGE bis-Tris gels with Bolt morpholineethanesulfonic acid (MES) SDS running buffer and transferred to a nitrocellulose membrane under wet conditions (Invitrogen). GST- and 3×FLAG-tagged proteins were detected with a rabbit polyclonal anti-GST antibody (1:2,500; Sigma-Aldrich) and a mouse monoclonal horseradish peroxidase (HRP)-conjugated anti-FLAG antibody (M2; 1:10,000; Sigma-Aldrich), respectively. Secondary anti-rabbit HRP-conjugated antibody was purchased from Invitrogen (1:5,000). Nitrocellulose membrane was then incubated with a peroxidase substrate (Clarity Western ECL substrate; Bio-Rad) and visualized with the ChemiDoc MP imaging system (Bio-Rad).

### Generation of CRISPR-Cas9 knockout cell lines.

To generate VPS39 knockout cells, suitable CRISPR/Cas9 target sites were identified within the first 5′-terminal exons present in all predicted mRNA splice variants of the VPS39 gene (GenBank accession no. NC_010443), and corresponding oligonucleotides were synthesized (Eurofins Genomics). The complementary oligonucleotide pair VPS39porc-gR3F and VPS39porc-gR3F, as well as VPS39porc-gR4F and VPS39porc-gR4F (Table S1), was phosphorylated and cloned into the BpiI-digested and dephosphorylated single guide RNA (sgRNA) and Cas9 nuclease expression vector pX330A-1x4neoR (our unpublished results). After verifying the correct sequence insertions, the resulting plasmids pX330-VPS39gR3neoR and pX330-VPS39gR4neoR were used to transfect WSL cells, and single Geneticin-resistant cell clones were selected with a medium containing 500 μg/mL G418 and tested for expression of the FLAG-tagged Cas9 nuclease by Western blot analyses of cell lysates using the monoclonal anti-FLAG M2 antibody (Sigma/Merck) as described previously (56).

Genomic DNA of Cas9-expressing cell clones was prepared (71) and tested for the presence of the desired sgRNA genes by PCR amplification and sequencing using primers X330GGR-F2 and X330GGR-R2 (Table S1), the BigDye Terminator v1.1 cycle sequencing kit, and an Applied Biosystems 3500 genetic analyzer (Thermo Fisher Scientific). Furthermore, genomic DNA of the cell clones was analyzed for CRISPR/Cas9-induced mutations by PCR amplification and sequencing of the targeted VPS39 gene regions using primers VPS39gR3T-136F and VPS39gR3T-950R, or VPS39gR4T-147F and VPS39gR4T-1003R, respectively (Table S1). Sequences were evaluated using the Geneious Prime 2021.0.1 software package (Biomatters [https://www.geneious.com]).

Similarly, a single cell clone was generated with an empty Cas9 nuclease expression vector pX330A-1x4neoR as a control (CTRL-KO). The absence of VPS39 in knockout cells was further confirmed by LC-MS/MS analysis. Lysates of WSL KO and control cells (100 μg) were prepared using the Thermo EasyPep Mini MS sample preparation kit (Thermo Scientific) according to the manufacturer’s instructions. Dried peptides were reconstituted in 0.1% formic acid to a final concentration of 100 ng/μL. Peptides corresponding to 200 ng protein were measured by LC-MS/MS and analyzed as described in “MS Data Analysis.”

### Data availability.

All mass spectrometry raw data and MaxQuant output tables were deposited in the ProteomeXchange Consortium (http://proteomecentral.proteomexchange.org) via the PRIDE partner repository (72) (identifier PXD035695).
