# Supplementary material for: CP204L Is a Multifunctional Protein of African Swine Fever Virus That Interacts with the VPS39 Subunit of the Homotypic Fusion and Vacuole Protein Sorting Complex and Promotes Lysosome Clustering
Source: J Virol. 2023 Feb 1;97(2):e01943-22. doi: 10.1128/jvi.01943-22 (PMC9972913; doi:10.1128/jvi.01943-22)
Supplement: Supplemental file 1 — Fig. S1 and S2 and Table S1. Download jvi.01943-22-s0001.pdf, PDF file, 0.1 MB [file jvi.01943-22-s0001.pdf]

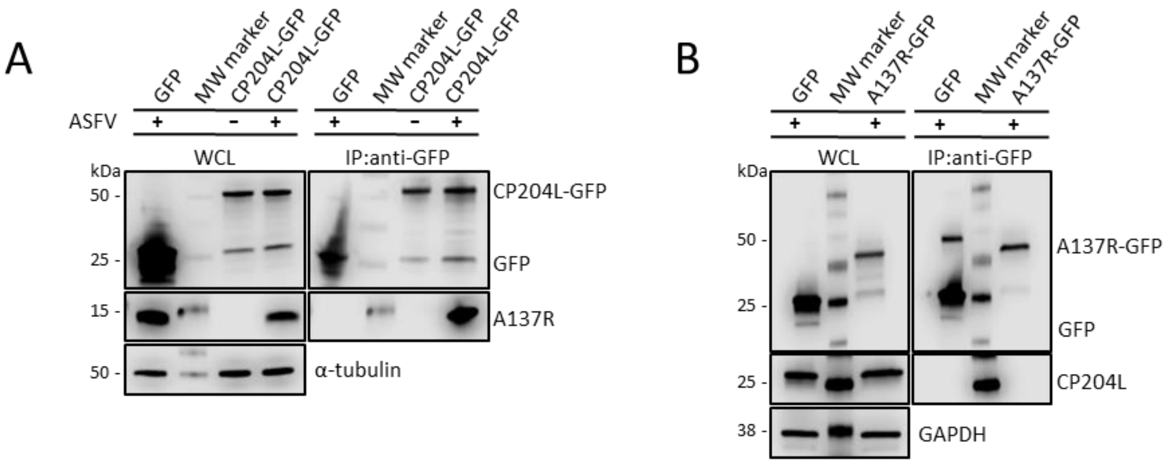

Fig S1. Reverse pulldown failed to confirm the interaction between ASFV CP204L and ASFV A137R.

(A) Co-immunoprecipitation of CP204L-GFP with A137R in ASFV-infected cells. Lysates from mock and ASFV-infected cells stably expressing CP204L-GFP or GFP alone were subjected to GFP-specific immunoprecipitation. Representative immunoblots of whole cell lysates (WCLs) and GFP-immunoprecipitates (IP) are shown. α-tubulin was used as a loading control in WCLs. (B) Reverse co-immunoprecipitation of GFP-A137R with CP204L in ASFV-infected cells. No interaction between GFP-A137R and CP204L was detected (no band was present in the IP fraction). GFP was used as a control. GAPDH was used as a loading control in WCLs.

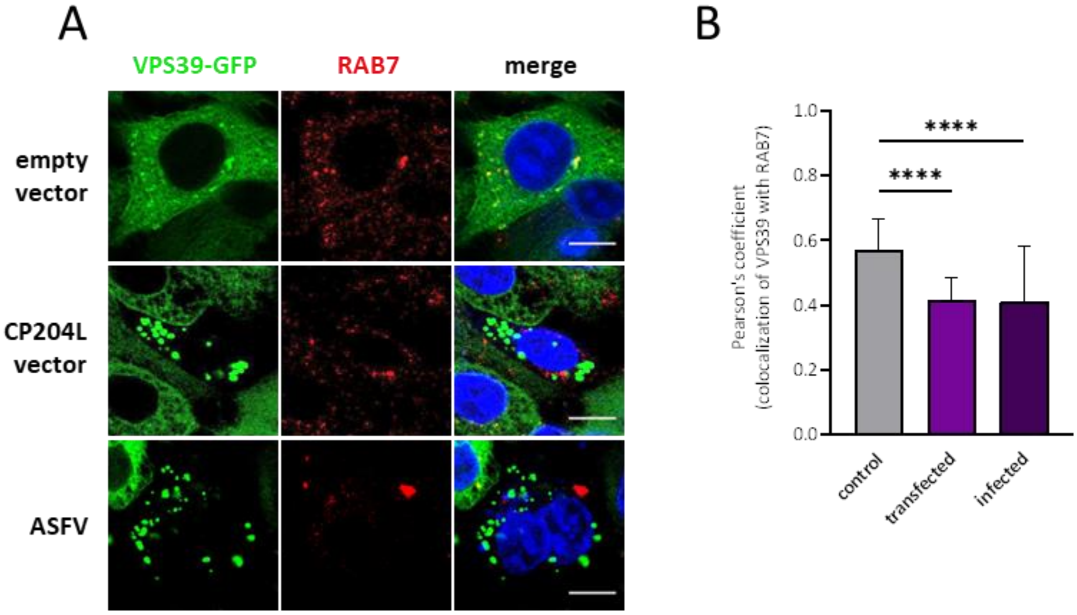

Fig S2. CP204L blocks VPS39 targeting to late endosomes.

(A) WSL cells stably expressing VPS39-GFP were observed by fluorescence confocal microscopy after transfection with CP204L expression construct or infection with ASFV. Cells were stained with an anti-Rab7 antibody, a late endosomal marker. Scale bars, 10 µm. (B) Quantification of the colocalization of VPS39 with late endosomes labeled by Rab7. Pearson's coefficient (mean ± SEM) from 25 cells in each group. **** $p < 0.0001$.

Table S1: Synthetic oligonucleotide primers

Name	DNA sequence
pCAG-F3	5’-GCTAACCATGTTCATGCCTTC-3’
ASFVp30CDS-R	5’-ACAGGATCCGCGATGTACGTCAGGTAGAAGC-3’
VPS39porc-gR3F	5’-*CACC*GCTCCAGCTGTTTTCCTGTT-3’
VPS39porc-gR3R	5’-*AAAC*AACAGGAAAACAGCTGGAGC-3’
VPS39porc-gR4F	5’-*CACC*GTTGAAATGTCAGTAGGTCG-3’
VPS39porc-gR4R	5’-*AAAC*CGACCTACTGACATTTCAAC-3’
X330GRR-F2	5’-ATGCTTACCGTAACTTGAAAG-3’
X330GRR-R2	5’-ATTTGTCTGCAGAATTGGCG-3’
VPS39gR3T-136F	5’-TAGGAATGGGATTGTTGGG-3’
VPS39gR3T-950R	5’-AATGTTGCATCCTTCACCC-3’
VPS39gR4T-147F	5’-ATTGTGCTTGCTTTGTTGG-3’
VPS39gR4T-1003R	5’-GTTTCAACTTTGGCTCACC-3’

Primers used for generation and characterization of ASFV CP204L expression constructs and of VPS39 knockout swine cells. Relevant restriction sites are underlined, and sequence overhangs for cloning into *Bpi*I-digested pX330A-1x4neoR are printed in Italics.
